# On the Sintering Behavior of Nb_2_O_5_ and Ta_2_O_5_ Mixed Oxide Powders

**DOI:** 10.3390/ma15145036

**Published:** 2022-07-20

**Authors:** Maureen P. Chorney, Kunal Mondal, Jerome P. Downey, Prabhat K. Tripathy

**Affiliations:** 1 Department of Materials Science and Engineering, Montana Technological University, Butte, MT 59701, USA; mchorney@mtech.edu (M.P.C.); jdowney@mtech.edu (J.P.D.); 2 Materials Science and Engineering Department, Energy and Environment Science and Technology Directorate, Idaho National Laboratory, Idaho Falls, ID 83415, USA; kunal.mondal@inl.gov; 3 Pyrochemistry & Molten Salt Systems Department, Fuel Cycle Science and Technology Division, Nuclear Science and Technology Directorate, Idaho National Laboratory, Idaho Falls, ID 83415, USA

**Keywords:** tantalum pentoxide, niobium pentoxide, energy materials, oxide precursor, sintering, green manufacturing

## Abstract

A mixed oxide system consisting of Nb_2_O_5_ and Ta_2_O_5,_ was subjected to annealing in air/hydrogen up to 950 °C for 1–4 h to study its sintering behavior. The thermogravimetric–differential scanning calorimetry (TGA–DSC) thermograms indicated the formation of multiple endothermic peaks at temperatures higher than 925 °C. Subsequently, a 30% Ta_2_O_5_ and 70% Nb_2_O_5_ (mol%) pellet resulted in good sintering behavior at both 900 and 950 °C. The scanning electron microscope (SEM) images corroborated these observations with necking and particle coarsening. The sintered pellets contained a 20.4 and 20.8% mixed oxide (Nb_4_Ta_2_O_15_) phase, along with Ta_2_O_5_ and Nb_2_O_5_, at both 900 and 950 °C, indicating the possibility of the formation of a solid solution phase. In situ high-temperature X-ray diffraction (XRD) scans also confirmed the formation of the ternary oxide phase at 6 and 19.8% at 890 and 950 °C, respectively. The Hume–Rothery rules could explain the good sintering behavior of the Ta_2_O_5_ and Nb_2_O_5_ mixed oxides. An oxide composition of 30% Ta_2_O_5_ and 70% Nb_2_O_5_ (mol%) and a sintering temperature of 950 °C appeared adequate for fabricating well-sintered oxide precursors for subsequent electrochemical polarization studies in fused salts.

## 1. Introduction

Transition metals and their alloys, compounds, and large complexes containing transition metals are widely used for a variety of purposes ranging from specialized high-temperature applications to more common uses, such as in catalysis, materials synthesis, photochemistry, biological systems, environment cleaning, and electronics. The fabrication of transition-metal-based functional oxides and hydroxides from aqueous metal-oxo cluster precursors and electrochemical routes has opened up numerous opportunities for the development of sustainable green chemistry. These suitable chemistries have led to the development of new materials for energy generation and storage, data storage, and many other potential applications. Although metal-oxo cluster chemistries, pertaining to group V and VI elements, have been widely reported, some of the issues pertaining to ligand dynamics, post-synthesis ligand exchange, and cluster stability have largely remained unresolved [1]. While the controlled electrochemical synthesis approach has limited benefits, researchers have been unable to resolve some of the difficulties related to synthesis because of the prevalence of incomplete solid-state diffusion kinetics [2]. Like the transition metal-oxo clusters, researchers have also developed rare-earth metal hexacyanoferrates for electrocatalysis and adsorbents for heavy ion removal applications [2,3,4,5,6,7]. In addition to the transition and rare-earth metal complexes, hexacyanoferrate simple oxides are also widely used in many technologies.

Many sectors use two of the group V oxides, Ta_2_O_5_ and Nb_2_O_5_. Because of its excellent dielectric properties, Ta_2_O_5_ is used in dynamic random access memory chips, field effect transistors, thin-film electroluminescence devices, biological and chemical sensors, antireflection coatings for silicon solar cells, charge-coupled devices, corrosion-resistant materials, optical wave guides, and thin-film resistors [8]. Nb_2_O_5_, on the other hand, exhibits strong redox abilities and unique Lewis and Brønsted acid sites, and as a result has been used for photocatalytic activities [9]. Another application of Nb_2_O_5_ has been in the fabrication of resistive random memory access devices because of the dependence of its dual electrical response of memory and threshold switching behaviors on oxygen contents resistive to random memory access devices [10]. Nb_2_O_5_ is routinely used in gas sensors, catalysis, optical and electrochromic devices, solid-state electrochemical devices, and biocompatible prostheses [11]. Because of the similarity in their chemical and structural properties, Ta_2_O_5_ and Nb_2_O_5_ mixed oxides have been used as coatings and thin films to either enhance the dielectric permittivity of Ta_2_O_5_ or the band gap of Nb_2_O_5_ [12]. Forming a mixed oxide phase, a solid solution of Nb_2_O_5_ and Ta_2_O_5_ (NbTaO_5_, (Ta_1−x_Nb_x_)_2_O_5_(Ta_1−x_Nb_x_)_2_O_5_, x = 0.02–0.07) is advantageous in terms of having a lower leakage current and enhanced dielectric permittivity in the device [12,13]. Fine (micron-sized) Nb_2_O_5_ and Ta_2_O_5_ powders formed reactive thermites and composites with nanosized aluminum, where the oxide mixture acted as the gasless oxidizer and the metal (aluminum) was a fuel [14].

Binary alloys of niobium and tantalum can serve as anticorrosion coatings (for CoCr alloys in the biomedical industry) [15] in the fabrication of high-entropy shape memory and superconducting alloys [16,17,18]. These applications employ a melting-cum-remelting process to make a homogeneous alloy from which the components are fabricated. In recent years, researchers have developed a novel electrochemical process to fabricate many metallic materials, both metals and alloys, from their oxide and mixed oxide intermediates. Although both Ta_2_O_5_ and Nb_2_O_5_, individually, have been successfully converted to tantalum and niobium, respectively, in molten salts [19,20,21], studies on the co-reduction of the mixed oxides in molten salts to form the binary (NbTa) alloy are absent in the literature. Both Nb_2_O_5_ and Ta_2_O_5_ were mixed with other oxides (TiO_2_, ZrO_2_, and HfO_2_) to form high-entropy alloys, consisting of titanium–niobium–tantalum–zirconium and titanium–niobium–tantalum–zirconium–hafnium, in a calcium chloride melt [22]. However, no studies appear to have reported on the formation of the binary alloys from their mixed oxide precursors. Three types of salts (LiCl-Li_2_O, CaCl_2_-CaO, and eutectic CaCl_2_-NaCl) have been used to electrochemically reduce metal oxides to their metallic constituent. Each electrolyte system offers a set of advantages and disadvantages. CaCl_2_ provides two distinct advantages: the relatively higher solubility of the oxide ions in calcium chloride and enhanced reduction kinetics, thereby decreasing the overall reduction time. The objective of the present study was to examine the co-reduction behavior of the mixed oxides in a calcium chloride melt. The experimental research was divided into two parts: (1) the preparation, evaluation, and characterization of mixed oxide precursor; (2) the electrochemical reduction of precursor materials prepared under a set of optimum conditions. The present manuscript describes the experimental results pertaining to the preparation and characterization of mixed oxide precursors. The experimental work consisted of the mixing and homogenization of Ta_2_O_5_ and Nb_2_O_5_, thermal analyses of the powder, the pelletization of the homogenized powder, the sintering of the mixed powder, a study of the powder’s morphology using a scanning electron microscope, and phase analyses of the heat-treated powders via room- and high-temperature X-ray diffraction.

## 2. Materials and Methods

### 2.1. Materials

High-purity and finely powdered tantalum pentoxide (Ta_2_O_5_, Sigma-Aldrich (St. Louis, MO, USA) 99.99% trace metals basis, <20 µm) and niobium pentoxide (Nb_2_O_5_, Sigma-Aldrich, 99.9% trace metals basis, −325 mesh) were used as the starting materials. Polyvinyl alcohol/[poly (vinyl butyral-co-vinyl alcohol-co-vinyl acetate)] (PVB/PVA), Sigma-Aldrich, average MW = 50,000–80,000 by gel permeation chromatography (GPC) and poly (ethylene glycol, PEG, Sigma-Aldrich, average MW = 200) were used as the binder to prepare the powder mixture. Finally, the powder mixture was homogenized in a ball miller for 4 h and the slurry was dried (under a heat lamp) over a period of ~36 h.

### 2.2. Equipment

A thermogravimetric analyzer (simultaneous TGA–DSC, SDT Q600, TA instruments, New Castle, DE, USA) performed the initial heat treatment of the milled (mixed) oxide powder. An MTI 1100X Series tube furnace (MTI corporation, Richmond, CA, USA) sintered the pelletized powder mixture. An X-ray diffraction unit with PDXL (Rigaku, Japan) and JADE software (MDI, Hibbing, MN, USA) programs analyzed the diffraction patterns. A small furnace containing a platinum tray and a scintillation detector was used to collect X-ray diffraction (XRD, Rigaku Ultima IV diffractometer) data. A D/teX Ultra detector (Rigaku, Tokyo, Japan) recorded the room-temperature XRD patterns of the sintered pellets. A scanning electron microscope (MIRA3 TESCAN SEM, TESCAN USA, Inc., Warrendale, PA, USA) with an energy-dispersive X-ray (EDS) analysis attachment was used to examine the sintered pellet morphologies.

### 2.3. Procedure

Calculated quantities of Ta_2_O_5_ and Nb_2_O_5_ powders were mixed and homogenized in an agate mortar and pestle. About 8–15 mg powders were placed in an alumina crucible, which in turn was loaded into the simultaneous TGA–DSC unit. The mixed powder was heated at 5 °C/min to a maximum temperature of 1150 °C to record the endo- and exothermic peaks. This heating was performed under a continuous argon flow. Depending on the thermogram results, a few powder compositions were formulated to record the exothermic and endothermic peaks and percentage mass loss. A laboratory hydraulic pressing unit was used to pelletize the milled powder. The powder was compacted into 13 mm dia. pellets in a steel die by applying around 29–29.6 MPa of pressure. The green pellets were subsequently loaded into an alumina boat and heated in air/hydrogen up to the desired temperatures (up to 950 °C) for fixed durations (1–4 h). The sintered pellets were subsequently evaluated with respect to their phase compositions and morphological features with a 10 °C/min heating rate to record the in situ high-temperature XRD data. The temperature controller was set up to hold at the set temperature for 5 min in order to have a sample with a uniform temperature across its surface. The detailed information pertaining to the preparation of samples and experimental procedure is described elsewhere [23].

## 3. Results and Discussion

### 3.1. TGA–DSC Studies

**Figure 1:**
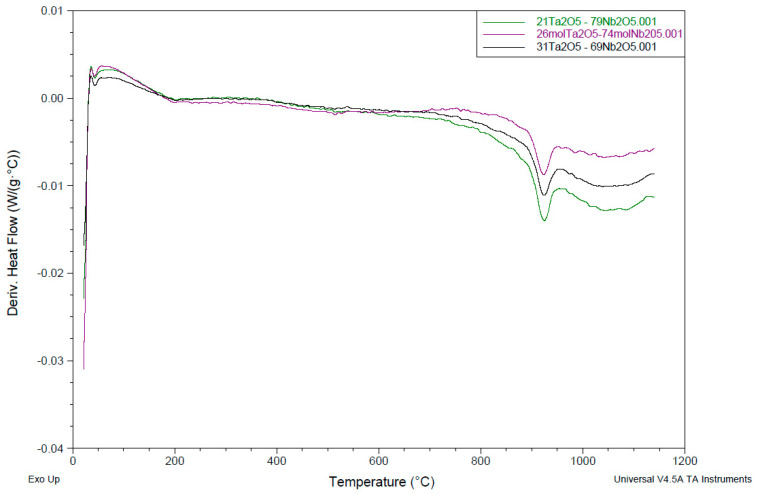
DSC scans of three Ta_2_O_5_-Nb_2_O_5_ compositions (green: 21 Ta_2_O_5_ to 79 Nb_2_O_5_; violet: 31 Ta_2_O_5_ to 69 Nb_2_O_5_; grey: 26 Ta_2_O_5_ to 74 Nb_2_O_5_; all concentrations are in mol%).

**Figure 2:**
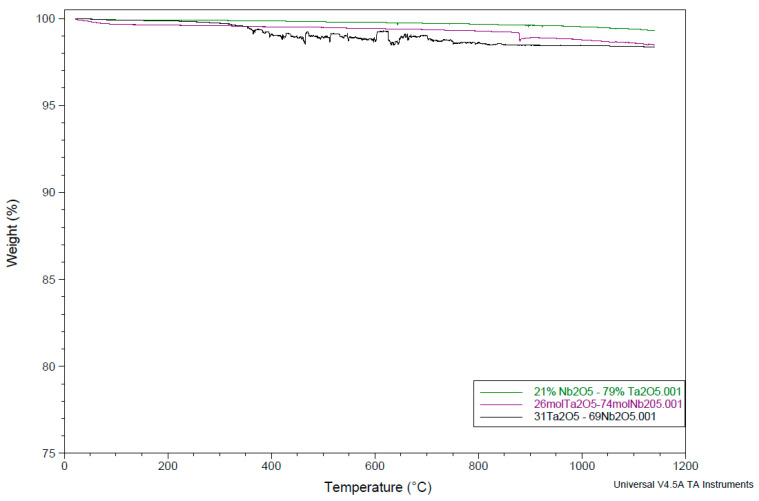
TGA scans of three Ta_2_O_5_-Nb_2_O_5_ compositions (green: 21 Ta_2_O_5_ to 79 Nb_2_O_5_; violet: 31 Ta_2_O_5_ to 69 Nb_2_O_5_; grey: 26 Ta_2_O_5_ to 74 Nb_2_O_5_; all concentrations are in mol%) showing weight loss (%).

A 50:50 (mass%) composition of the mixed powder showed three endothermic peaks, at 925.62 °C, 1006.1 °C, and 1036.66 °C, respectively. The Ta_2_O_5_-Nb_2_O_5_ binary phase diagram indicates the formation of a solid solution in a composition with about 26 mol% Ta_2_O_5_ [24]. This is why it was decided to investigate a few compositions around 26 mol% to observe the formation of any solid solution and the ternary oxide composition that might influence the subsequent electrochemical reduction step. A composition of 26 to 74 mol% Ta_2_O_5_ and Nb_2_O_5_, respectively, showed a single endothermic peak at 923.75 °C. Similarly, two more compositions of 21 to 79 mol% and 31 to 69 mol% Ta_2_O_5_-Nb_2_O_5_ showed endothermic peaks at just one temperature (924.54 °C) (Figure 1). The TGA indicated insignificant weight loss values (1.48–2.04%) (Figure 2). These studies indicated that perhaps a temperature of around or higher than 925 °C is needed to obtain an adequately sintered pellet.

### 3.2. Morphology of the Sintered Pellets

**Figure 3:**
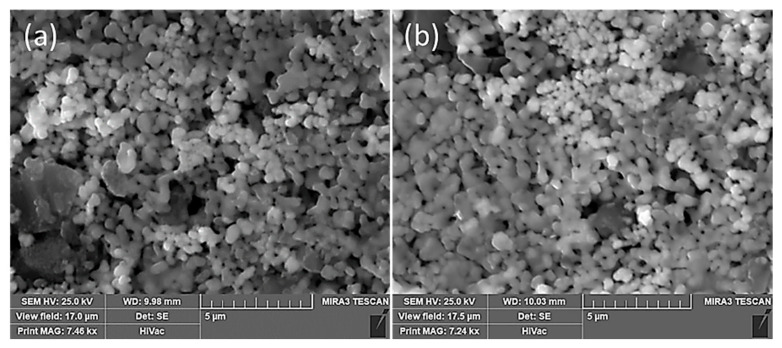
Scanning electron microscope images of sintered pellets: (**a**) 900 °C; (**b**) 950 °C.

Based on the TGA–DSC studies, a 30 mol% Ta_2_O_5_ to 70 mol% Nb_2_O_5_ composition was used to prepare the mixed oxide pellets from the homogenized powder. The green pellets were subsequently heated at two different temperatures (900 and 950 °C, respectively) to observe the overall sintering behavior of the mixed powders. Figure 3 shows scanning electron microscope images of the pellets sintered at 900 and 950 °C. Both pellets showed very good sintering behavior consisting of necking (initial sintering stage) and reductions in porosity and particle size coarsening. As expected, the heat-treated pellet at 950 °C showed a better degree of sintering as compared to the pellet, which was sintered at 900 °C. Some of the scattered darker pieces were alumina, which may have come from the grinding media. The EDS analysis, as expected, showed tantalum, niobium, and oxygen as major peaks along with traces of aluminum and carbon due to the contamination and use of a carbon tape. Based on the morphological features, precursor materials were prepared at 950 °C for their subsequent conversion to the binary (metallic) alloy.

### 3.3. Phase Identification: Room-Temperature X-ray Diffraction Patterns

The XRD data indicated that both oxide phases (Ta_2_O_5_ and Nb_2_O_5_) were present. In addition to the two binary phases, the formation of a mixed oxide phase (Nb_4_Ta_2_O_15_) could also be identified (Equation (1)):

Ta_2_O_5_ + 2 Nb_2_O_5_ = Nb_4_Ta_2_O_15_, (1)

Contrary to these findings, Fazio et al. [25] could not detect any ternary oxide when they doped amorphous Ta_2_O_5_ thin films with Nb_2_O_5_ and sintered the resultant product in air up to 900 °C for 10 h. According to these authors, both Ta_2_O_5_ and Nb_2_O_5_ are quite stable when forming any ternary oxides. They concluded that in the absence of any ternary oxide phase, the dopant (Nb_2_O_5_) acted as an amorphizing agent that increased the thermal stability of the Ta_2_O_5_ thin films [25]. The new phase (Nb_4_Ta_2_O_15_) did not have any reported reference intensity ratio (RIR) values. This was why the diffraction patterns were analyzed with the JADE software to estimate the RIR values. In order to obtain the relative weight percentages of the compounds within the sample, it is necessary to assign a RIR value to derive the quantitative information. Three RIR values, 2.8 (Nb_2_O_5_), 8.2 (Ta_2_O_5_), and their mean (5.5), were selected. Table 1, Table 2, Table 3, Table 4, Table 5 and Table 6 list the quantitative phase analyses of the pellets sintered at 900 and 950 °C with three sets of RIR values in each case.

With a lower RIR value, the quantity of niobium tantalum oxide was as high as 56.1% of the total phase composition. A relatively larger peak with a small RIR value indicates a larger quantity of that particular phase. In comparison, a relatively larger peak with a larger RIR value will result in a smaller quantity of the phase of interest as the larger RIR value indicates that the peak of the particular phase is naturally higher than that of the reference standard (corundum). The largest RIR indicated that 20.4% of the sample consisted of the ternary oxide phase.

A similar phase distribution was observed while analyzing a pellet sintered at 950 °C. Although the overall quantity of niobium tantalum oxide (RIR = 2.8) was slightly lower than the value at 900 °C, at 42.2% compared to 56.1%, the 5.5 and 8.2 RIR values produced similar results at both sintering temperatures. The amounts of mixed oxide phases at 900 and 950 °C for an RIR value of 5.5 were 28 and 26.2%, respectively. Similarly, for an RIR value of 8.2, the mixed oxide phases were 20.4 and 20.8% at 900 and 950 °C, respectively.

The quantitative phase analysis data from the room-temperature XRD scans indicated mixed oxide compositions of 20.4 and 20.8% at 900 and 950 °C, respectively. The binary phase diagram indicated the possibility of the formation of a solid solution in these compositions.

### 3.4. Phase Identification: High-Temperature X-ray Diffraction

**Figure 4:**
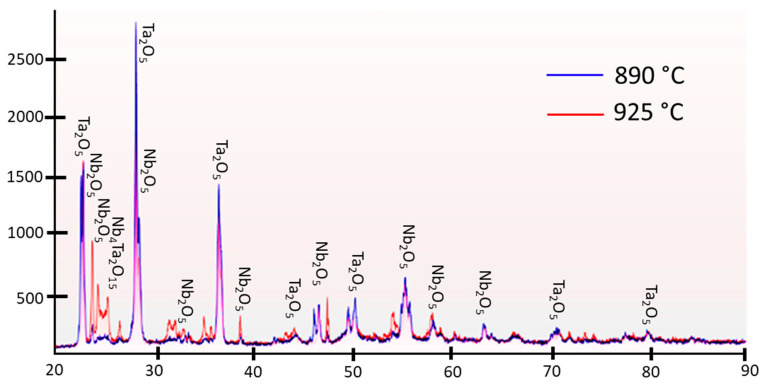
Overlay of the diffraction patterns recorded at two different temperatures (890 and 925 °C, respectively) for a 70 mol% Ta_2_O_5_ and 30 mol% Nb_2_O_5_ pellet, respectively.

In situ XRD patterns were recorded at four different temperatures (25, 890, 925, and 950 °C, respectively). The quantitative data for different RIR values are presented in Table 7, Table 8 and Table 9. As expected, the fraction of the mixed oxide phase was higher at 950 °C (19.8–34.4%) than at 890 °C (6.0–13.2%). A comparison between the room- and high-temperature XRD data for the mixed oxide phase indicated an interesting pattern. While there was no change between the values obtained at 900 and 950 °C (at room temperature), the fraction registered a somewhat lower value at 950 °C (42.2%) than at 900 °C (56.1%). Figure 4 shows an overlay of the diffraction patterns recorded at 890 °C and 925 °C, respectively. These results indicated a definitive trend in the mixed oxide content, with an increase in temperature from 890 to 925 °C. Although the initial percentages of tantalum pentoxide and niobium pentoxide appeared to be skewed at 25 °C (Table 7, Table 8 and Table 9), the subsequent values showed consistencies at other temperatures, with the values decreasing as the formation of the Nb_4_Ta_2_O_15_ phase increased. A minimal amount of mixed oxide was detected at 890 °C, but the composition changed dramatically when the temperature was increased by 35 °C. The amount of Nb_4_Ta_2_O_15_ decreased as the temperature was increased to 950 °C, which could have resulted because of the presence of metastable phases at 925 °C, which became unstable at higher temperatures. Alternatively, the decrease might have happened as the result of an error in the RIR value.

Mohanty et al. [24] investigated the phase equilibria in the mixed oxide system in a bid to determine the discrepancies reported in the literature. They prepared the mixed oxide systems by heating the mixtures of Ta_2_O_5_ and Nb_2_O_5_ in oxygen, argon, and an argon–air mixture (80% Ar–20% oxygen) to determine the effects of such treatments on the stoichiometry and structure of the base materials. They did observe the formation of α-Ta_2_O_5_ phase and 2Nb_2_O_5_.Ta_2_O_5_ compound at 1435 °C when the Ta_2_O_5_ content was higher than 36% [26]. They also reported the formation of α-Nb_2_O_5_ and β-Ta_2_O_5_ at a slightly lower temperature range (1200–1300 °C) [24]. Some other authors have also reported similar studies [27]. Mohanty et al. also reported high-temperature XRD measurements. Their studies indicated the formation of a two-phase region (α-Ta_2_O_5_ + β-Ta_2_O_5_) in the composition range of 50–100% Ta_2_O_5_ [24]. 

As stated earlier, the focus in the present investigation was to prepare a stable mixed oxide precursor (which should not undergo disintegration during the subsequent electrochemical polarization in calcium chloride melt at ~800 °C) and not the detailed phase analysis. Unlike in other (reported) studies, where the mixed oxide compositions were kept at 1000 °C for a duration of 24 h [24], in the present studies the temperature and duration were <1000 °C and up to 4 h, respectively. Like other studies, the present study alco confirmed the presence of the ternary oxide (Nb_4_Ta_2_O_15_) along with the other two oxides (Ta_2_O_5_ and Nb_2_O_5_) at all temperatures, albeit after heating for a relatively fewer number of hours. 

## 4. Conclusions

Mixed oxides, containing Ta_2_O_5_ and Nb_2_O_5_ in different molar ratios, were subjected to thermal treatment in a TGA–DSC setup up to a maximum of 1150 °C. The recorded thermograms indicated sintering behavior at temperatures over 850 °C. The morphology of the sintered powders indicated good sintering behavior at or above 900 °C for a 30 mol% Ta_2_O_5_ to 70 mol% Nb_2_O_5_ composition. The Hume–Rothery rules (identical ionic radii, valence states, electronegativity, and crystal structure) could explain the good sintering behavior of the Ta_2_O_5_ and Nb_2_O_5_ mixed oxides. Furthermore, 950 °C is adequate to prepare the mixed oxide precursors (30 mol% Ta_2_O_5_ to 70 mol% Nb_2_O_5_) with good mechanical integrity. Both the room- and high-temperature XRD studies indicated the formation of a ternary oxide phase. The quantitative determination of the mixed oxide phase is dependent on the relative intensity response (RIR) value. The sintered pellets, upon immersion into a pool of molten calcium chloride at 850 °C, did not form any surface. Two specific characteristics (percentage open porosity and good mechanical strength) were observed to be the critical parameters for the preparation of the mixed oxide precursors for their subsequent conversion to the binary metallic alloys. 

## Figures and Tables

**Table 1:** Quantitative XRD (phase) analysis results (whole pattern fitting (WPF) combined with RIR) of a 70 mol% Nb_2_O_5_ to 30 mol% Ta_2_O_5_ pellet at 900 °C (Nb_4_Ta_2_O_15_, RIR = 2.8).

Phase Name	Formula	RIR	DB Card No.	Content	Total Ta_x_Nb_y_O_z_
Tantalum Oxide	Ta_2_O_5_	8.20	01-025-0922	13.6	
Niobium Oxide	Nb_2_O_5_	2.79	01-071-1169	30.3	
Niobium Tantalum Oxide	Nb_4_Ta_2_O_15_	2.80	01-071-4513	56.1	
				100.0	56.1

**Table 2:** Quantitative XRD (phase) analysis results (WPF, combined with RIR) of a 70 mol% Nb_2_O_5_ to 30 mol% Ta_2_O_5_ pellet at 900 °C (Nb_4_Ta_2_O_15_, RIR = 5.5).

Phase Name	Formula	RIR	DB Card No.	Content	Total Ta_x_Nb_y_O_z_
Tantalum Oxide	Ta_2_O_5_	8.20	01-025-0922	25.2	
Niobium Oxide	Nb_2_O_5_	2.79	01-071-1169	46.8	
Niobium Tantalum Oxide	Nb_4_Ta_2_O_15_	5.50	01-071-4513	28.0	
				100.0	28.0

**Table 3:** Quantitative XRD (phase) analysis results (WPF, combined with RIR) of a 70 mol% Nb_2_O_5_ to 30 mol% Ta_2_O_5_ pellet at 900 °C (Nb_4_Ta_2_O_15_, RIR = 8.2).

Phase Name	Formula	RIR	DB Card No.	Content	Total Ta_x_Nb_y_O_z_
Tantalum Oxide	Ta_2_O_5_	8.20	01-025-0922	27.7	
Niobium Oxide	Nb_2_O_5_	2.79	01-071-1169	51.9	
Niobium Tantalum Oxide	Nb_4_Ta_2_O_15_	8.20	01-071-4513	20.4	
				100.0	20.4

**Table 4:** Quantitative XRD (phase) analysis results (WPF, combined with RIR) of a 70 mol% Nb_2_O_5_ to 30 mol% Ta_2_O_5_ pellet at 950 °C (Nb_4_Ta_2_O_15_, RIR = 2.8).

Phase Name	Formula	RIR	DB Card No.	Content	Total Ta_x_Nb_y_O_z_
Tantalum Oxide	Ta_2_O_5_	8.20	01-025-0922	21.9	
Niobium Oxide	Nb_2_O_5_	2.79	01-071-1169	35.8	
Niobium Tantalum Oxide	Nb_4_Ta_2_O_15_	2.80	01-071-4513	42.2	
				99.9	42.2

**Table 5:** Quantitative XRD (phase) analysis results (WPF, combined with RIR) of a 70 mol% Nb_2_O_5_ to 30 mol% Ta_2_O_5_ pellet at 950 °C (Nb_4_Ta_2_O_15_, RIR = 5.5).

Phase Name	Formula	RIR	DB Card No.	Content	Total Ta_x_Nb_y_O_z_
Tantalum Oxide	Ta_2_O_5_	8.20	01-025-0922	29.6	
Niobium Oxide	Nb_2_O_5_	2.79	01-071-1169	44.0	
Niobium Tantalum Oxide	Nb_4_Ta_2_O_15_	5.50	01-071-4513	26.3	
				99.9	26.3

**Table 6:** Quantitative XRD (phase) analysis results (WPF, combined with RIR) of a 70 mol% Nb_2_O_5_ to 30 mol% Ta_2_O_5_ pellet at 950 °C (Nb_4_Ta_2_O_15_, RIR = 8.2).

Phase Name	Formula	RIR	DB Card No.	Content	Total Ta_x_Nb_y_O_z_
Tantalum Oxide	Ta_2_O_5_	8.20	01-025-0922	24.9	
Niobium Oxide	Nb_2_O_5_	2.79	01-071-1169	54.3	
Niobium Tantalum Oxide	Nb_4_Ta_2_O_15_	8.20	01-071-4513	20.8	
				100.0	20.8

**Table 7:** Quantitative phase analysis results (WPF) from the high-temperature XRD scans of a 30 mol% Ta_2_O_5_ to 70 mol% Nb_2_O_5_ pellet (Nb_4_Ta_2_O_15_ RIR = 2.8).

°C	Phase Name	Formula	RIR	PDF No	Content	Total Ta_x_Nb_y_O_z_
25	Tantalum Oxide	Ta_2_O_5_	8.20	00-025-0922	60.3	
	Niobium Oxide	Nb_2_O_5_	2.79	00-030-0873	39.7	
					100.0	—
890	Tantalum Oxide	Ta_2_O_5_	8.20	00-025-0922	25.4	
	Niobium Oxide	Nb_2_O_5_	2.79	00-030-0873	61.4	
	Niobium Tantalum Oxide	Nb_4_Ta_2_O_15_	2.80	00-015-0114	13.2	
					100.0	13.2
925	Tantalum Oxide	Ta_2_O_5_	8.20	00-025-0922	31.0	
	Niobium Oxide	Nb_2_O_5_	2.79	00-030-0873	50.7	
	Niobium Tantalum Oxide	Nb_4_Ta_2_O_15_	2.80	00-015-0114	18.3	
					100.0	18.3
950	Tantalum Oxide	Ta_2_O_5_	8.20	00-025-0922	34.7	
	Niobium Oxide	Nb_2_O_5_	2.79	00-030-0873	45.5	
	Niobium Tantalum Oxide	Nb_4_Ta_2_O_15_	2.80	00-015-0114	19.8	
					100.0	19.8

**Table 8:** Quantitative phase analysis results (WPF) from the high-temperature XRD scans of a 30 mol% Ta_2_O_5_ to 70 mol% Nb_2_O_5_ pellet (Nb_4_Ta_2_O_15_ RIR = 5.5).

°C	Phase Name	Formula	RIR	PDF No	Content	Total Ta_x_Nb_y_O_z_
25	Tantalum Oxide	Ta_2_O_5_	8.20	00-025-0922	60.3	
	Niobium Oxide	Nb_2_O_5_	2.79	00-030-0873	39.7	
					100.0	—
890	Tantalum Oxide	Ta_2_O_5_	8.20	00-025-0922	36.2	
	Niobium Oxide	Nb_2_O_5_	2.79	00-030-0873	55.8	
	Niobium Tantalum Oxide	Nb_4_Ta_2_O_15_	5.50	00-015-0114	8.0	
					100.0	8.0
925	Tantalum Oxide	Ta_2_O_5_	8.20	00-025-0922	42.3	
	Niobium Oxide	Nb_2_O_5_	2.79	00-030-0873	18.0	
	Niobium Tantalum Oxide	Nb_4_Ta_2_O_15_	5.50	00-015-0114	39.7	
					100.0	39.7
950	Tantalum Oxide	Ta_2_O_5_	8.20	00-025-0922	41.2	
	Niobium Oxide	Nb_2_O_5_	2.79	00-030-0873	24.3	
	Niobium Tantalum Oxide	Nb_4_Ta_2_O_15_	5.50	00-015-0114	34.4	
					99.9	34.4

**Table 9:** Quantitative phase analysis results (WPF) from the high-temperature XRD scans of a 30 mol% Ta_2_O_5_ to 70 mol% Nb_2_O_5_ pellet (Nb_4_Ta_2_O_15_ RIR = 8.2).

°C	Phase Name	Formula	RIR	PDF No	Content	Total Ta_x_Nb_y_O_z_
25	Tantalum Oxide	Ta_2_O_5_	8.20	00-025-0922	60.3	
	Niobium Oxide	Nb_2_O_5_	2.79	00-030-0873	39.7	
					100.0	—
890	Tantalum Oxide	Ta_2_O_5_	8.20	00-025-0922	36.2	
	Niobium Oxide	Nb_2_O_5_	2.79	00-030-0873	55.8	
	Niobium Tantalum Oxide	Nb_4_Ta_2_O_15_	8.20	00-015-0114	8.0	
					100.0	6.0
925	Tantalum Oxide	Ta_2_O_5_	8.20	00-025-0922	42.3	
	Niobium Oxide	Nb_2_O_5_	2.79	00-030-0873	18.0	
	Niobium Tantalum Oxide	Nb_4_Ta_2_O_15_	8.20	00-015-0114	39.7	
					100.0	26.5
950	Tantalum Oxide	Ta_2_O_5_	8.20	00-025-0922	41.2	
	Niobium Oxide	Nb_2_O_5_	2.79	00-030-0873	24.3	
	Niobium Tantalum Oxide	Nb_4_Ta_2_O_15_	8.20	00-015-0114	34.4	
					100.0	21.1

## Data Availability

Not applicable.

## Disclaimer

The information was prepared as an account of the work sponsored by an agency of the U.S. government. Neither the U.S. government nor any agency thereof, nor any of their employees, makes any warranty, expressed or implied, or assumes any legal liability or responsibility for the accuracy, completeness, or usefulness of any information, apparatus, product, or process disclosed, or represents that its use would not infringe on privately owned rights. References herein to any specific commercial product, process, or service by trade name, trademark, manufacturer, or otherwise does not necessarily constitute or imply its endorsement, recommendation, or favoring by the U.S. government or any agency thereof. The views and opinions of the authors expressed herein do not necessarily state or reflect those of the U.S. government or any agency thereof.
